# Estimation of potential cost-savings related to the implementation of perioperative hemodynamic goal-directed therapy

**DOI:** 10.1186/cc12134

**Published:** 2013-03-19

**Authors:** T Simon, G Marx

**Affiliations:** 1 RWTH University Hospital Aachen, Germany

## Introduction

Many studies have demonstrated the ability of perioperative hemodynamic goal-directed therapy (pGDT) to decrease postoperative morbidity in patients undergoing medium-to-high-risk surgery [1]. As a result, pGDT may be a cost-saving strategy. Our goal was to provide an estimation of potential cost-savings based on recent literature.

## Methods

The largest and most recent meta-analysis [1] on pGDT was used to estimate what could be the reduction of postoperative morbidity if pGDT was to be adopted. Costs related to the treatment of patients developing at least one (1+) postoperative complication were obtained from two recent US [2] and Swiss [3] publications. Potential cost-savings related to the adoption of pGDT were calculated according to the actual morbidity rate, assuming 0% pGDT use so far, and 100% compliance rate after implementation.

## Results

The 2011 meta-analysis [1] of 29 RCTs (4,805 patients) showed that pGDT is associated with a reduction in the rate of patients developing 1+ postoperative complications with odd ratios ranging between 0.35 and 0.55. Importantly, these odd ratios were not related to the morbidity rates. In the US publication [2], extra costs for treating patients with 1+ complication were $17,949. In the Swiss (CH) publication [3], they were $34,446. See Table 1.

**Table 1:** 

Actual morbidity rate no pGDT (%)	10	20	30	40	50	60
Expected morbidity rate pGDT (%)	3.5 to 5.5	7 to 11	11 to 17	14 to 22	18 to 28	21 to 33
Expected cost reduction/US patient ($)	808 to 1,167	1,615 to 2,333	2,423 to 3,500	3,231 to 4,667	4,039 to 5,833	4,846 to 7,000
Expected cost reduction/CH patient ($)	1,550 to 2,239	3,100 to 4,478	4,478 to 6,545	6,200 to 8,956	7,578 to 11,023	9,300 to 13,434

## Conclusion

Depending on the pre-implementation morbidity rate, the degree of pGDT-induced morbidity reduction and the country, cost-savings ranged between $808 and $13,434 per patient. This large variability suggests that local/hospital estimations are desirable before starting pGDT implementation. These tailored evaluations would also allow more precise cost-saving estimations by taking into account the actual and expected pGDT compliance rates.
